# Antenatal group-based psychoeducation to improve postpartum depression literacy in primary health care institutions in Ethiopia: a cluster randomized controlled trial

**DOI:** 10.3389/fpsyt.2025.1548356

**Published:** 2025-04-17

**Authors:** Marta Tessema, Muluemebet Abera, Zewdie Birhanu

**Affiliations:** ^1^ School of Midwifery, Institute of Health, Jimma University, Jimma, Ethiopia; ^2^ Department of Population and Family Health, Institute of Health, Jimma University, Jimma, Ethiopia; ^3^ Department of Health, Behavior and Society, Institute of Health, Jimma University, Jimma, Ethiopia

**Keywords:** depression, postpartum, randomized controlled trial, health literacy, mental health, primary health care, maternal-child health services

## Abstract

**Background:**

Postpartum depression literacy assists mothers in recognizing and preventing postpartum depression (PPD). So, this study assessed the effectiveness of antenatal group psychoeducation in improving PPD literacy among pregnant women in Jimma, Ethiopia.

**Methods:**

A cluster randomized controlled trial was conducted from 28 March to 1 December 2022 involving 32 non-adjusted health centers that were randomized into two groups (16 health centers for each arm). The study enrolled 550 participants who scored (0–9) on the Patient Health Questionnaire-9. The intervention group received standard antenatal care and group psychoeducation, while the control group received only standard care. Postpartum depression literacy was assessed in face-to-face interviews at 12–20 weeks gestation and 6 weeks postpartum using the PPD literacy scale. Higher scores indicated higher literacy. An intention-to-treat analysis was used. Demographic factors were compared between groups using χ² and independent sample t-tests, indicating no significant differences except for educational status and parity. General linear models and mixed-effect models assessed intervention effects and outcome variable predictors, respectively.

**Results:**

The overall response rate was 92.9%. The study demonstrated a significant difference between groups regarding the overall mean PPD literacy score (intervention, 3.75 ± 0.46; control, 3.48 ± 0.46; ηp² = 0.07), ability to recognize PPD (intervention, 4.30 ± 0.64; control, 3.94 ± 0.75; ηp² = 0.06), knowledge of risk factors and causes (intervention, 4.03 ± 0.69; control, 3.67 ± 0.70; ηp² = 0.05), and access to PPD information (intervention, 3.28 ± 1.25; control, 2.01 ± 1.13; ηp ²= 0.21) at p = 0.001, with marginal significance regarding self-care activities (intervention 4.37 ± 0.54, control 4.26 ± 0.50, ηp² = 0.01, p = 0.051). Conversely, there were no significant differences in knowledge of professional help (intervention 2.97 ± 1.13, control 2.83 ± 0.80, p² = 0.00, p = 0.303), beliefs regarding professional help (intervention 2.67 ± 0.89, control 2.50 ± 0.72, p² = 0.01, p = 0.063), and attitudes toward PPD recognition and help seeking (intervention 3.91 ± 1.02, control 3.91 ± 1.02, p² = 0.00, p = 0.586). Moreover, partner emotional support (AOR = 0.1, 95% CI = 0.02–0.17), maladaptive coping [AOR = −0.14, 95% CI = −0.22–(−0.07)], and multiparty [AOR = −0.15, 95% CI = −0.22–(−0.08)] showed significant associations with overall PPD literacy score.

**Conclusions:**

The study showed that antenatal group psychoeducation had a moderate effect on overall PPD literacy score. However, further research is required to effectively change negative attitudes toward seeking formal help.

**Clinical Trial Registration:**

https://pactr.samrc.ac.za/, identifier PACTR 202203616584913.

## Introduction

Postpartum depression affects as many as 69.9% of mothers in Sub-Saharan Africa (1) and up to 33.8% of mothers in Ethiopia (2). This condition can have serious physical and psychological consequences for the mother, the child, and the family (3). Research shows that the lack of knowledge about the signs and symptoms of depression and treatment possibilities has been a major help-seeking barrier (4, 5). Postpartum depression literacy is a specialized form of mental health literacy that assists perinatal women in recognizing, managing, and preventing PPD. It focuses on the ability to recognize PPD, knowledge, and beliefs about risk factors, self-help strategies, professional help, and treatment options; attitudes that promote recognition and appropriate help seeking; and understanding of how to seek mental health information (6).

Research on PPD literacy among perinatal women is limited globally, with nearly all of these studies taking place in high-income countries. The available evidence indicates disparities in PPD literacy across nations and with specific focus areas or subscales (5). In recent years, there have been improvements in PPD literacy in high-income countries (7, 8). However, in low- and middle-income countries, evidence still shows a lack of knowledge and negative attitudes toward all aspects of PPD literacy (9, 10). Most studies have reported that mothers often struggle to differentiate between depression and anxiety, pregnancy-related symptoms, and baby blues. There is a failure to recognize PPD as a legitimate issue, accompanied by stigmatizing attitudes and beliefs toward PPD, such as: “It’s normal to have PPD,” “Mothers know how to look after a baby,” and “PPD comes and goes by itself.” Furthermore, many believe that social and cultural factors contribute to PPD leading to prefer informal sources of help over professional treatment (5, 7). Enhancing PPD literacy among mothers is crucial in facilitating preventive measures against PPD.

Available evidence shows that women with good PPD literacy better recognize depression and assess their mental state more appropriately (5, 7). The same study conducted on low-income women revealed the same finding that shows improvement in mental health care utilization of new mothers following PPD literacy (5, 9, 10). Furthermore, PPD literacy contains healthy lifestyle and self-help strategies that help to prevent and cope with stress and decrease the risk of depression.

Perinatal depression is prevalent in Ethiopia and significantly impacts the physical and psychological well-being of the mother, child, and family (11–13). Evidence indicates that mothers have low help-seeking behaviors (12, 14), which comes from inadequate perinatal depression knowledge at both the community and administrative levels, coupled with societal norms and insufficient governmental prioritization (12, 14, 15). As a result, there is no health education and counseling targeted to maternal mental health problem prevention or treatment options (12, 14–16). In Ethiopia, still, greater priority has been assigned to the physical health of the mothers in preventing pregnancy-related complications and deaths. The birth preparedness and complication readiness plan provides this care focusing primarily on the physical health of the women while neglecting their emotional well-being (16). So, the first step that helps to resolve this problem and prevent PPD is to increase mothers’ PPD literacy. So, this study tried to assess the effectiveness of antenatal group psychoeducation intervention in improving the PPD literacy of postnatal mothers.

Psychoeducation is one of the strategies that can assist people in comprehending various aspects of mental health, including prevention, treatment, and rehabilitation (17, 18). It is a flexible and easily implemented mental health intervention by maternal healthcare providers (18, 19) and helps to overcome attendance barriers in relation to time constraints (19). It can be administered individually or in groups to clients with similar types of problems and their families (17). Evidence indicates that all pregnant women are at risk of perinatal depression, and nearly all risk factors are shared by all mothers (20). So, psychoeducation is the best option of intervention to increase the knowledge of perinatal women and their families about PPD. Hence, this cluster randomized controlled trial tried to assess the effectiveness of antenatal group-based psychoeducation in improving PPD literacy of postnatal mothers in maternal healthcare facilities.

## Materials and methods

### Study design, area, and period

A two-arm, cluster randomized controlled trial was conducted in primary healthcare institutions in Jimma, Oromia Regional State, southwestern Ethiopia. The unit of randomization was health centers. The primary rationale for implementing a cluster-randomized controlled trial was that a component of this study has already been utilized at the facility level, hence allowing for the mitigation of information contamination. The findings were reported per the CONSORT reporting criteria (see **Supplementary File 1**). In the zone, there are more than 9 hospitals, 122 health centers, and 548 health posts. Women typically receive maternal healthcare services from primary healthcare units, which are primarily served by midwives and nurses (21). Maternal healthcare services in these facilities encompass comprehensive reproductive healthcare, including screening, prevention, education, and counseling, as well as maternal and fetal assessments for the prevention of common pregnancy problems and complications. Furthermore, the care includes treatment and referral services for pregnancy outcomes and complications. Nonetheless, these facilities do not provide teaching, counseling, or screening services explicitly aimed at maternal mental health (15). The study was carried out from 28 March to 1 December 2022.

### Population

All pregnant women, 12–20 weeks of gestational age attending antenatal care in the study area, were the source population, whereas all systematically selected and eligible pregnant women, 12–20 weeks of gestation, were the study population.

### Inclusion

The study included 32 non-adjusted health centers (clusters) that had comparable baseline characteristics, and maternal healthcare services were provided by female nurses and midwives who were aware of the local language and cultures and had at least 6 months of work experience. Similarly, pregnant mothers, 12–20 weeks of gestational age and non-depressed pregnant women who scored 0–9 on the Patient Health Questionnaire-9, were enrolled in the study (22).

### Exclusion criteria

The study also excluded pregnant women who met the above two criteria (gestational age and PPD status), but were severely ill, on treatment for a prior mental illness, or had hearing impairments.

### Sample size determination and sampling procedure

The study includes three outcome variables (PPD, PPD literacy, and social support). The sample sizes were calculated for all outcome variables, and the largest sample size was used as the final sample size based on the following assumptions. The proportion of PPD is 21.9% (13) with a 12% effect size (0.12), 32 clusters accessible (16 clusters in each arm), with a 95% CI and 80% power, and an intra-cluster correlation coefficient of 0.03558 (23). The normal approximation, G-power, a two-sample proportion comparison, and the design effect (design effect = 1 + p (m − 1) = 1.7) were employed. Additionally, adjustments were made for power, non-response rate, and the predetermined number of clusters. The sample size was calculated based on a fixed number of clusters (24, 25). The average cluster size for cluster randomization was 20, yielding a total sample size of 640 pregnant women (320 per group). The participant was sampled by using a systematic sampling technique. By taking three consecutive monthly reports of each health center (cluster), the average monthly load of antenatal care with specified gestational age was estimated. Finally, every two pregnant women (K = 2) were selected until the final sample size was met.

### Randomization and blinding

The units of randomization were health centers (clusters). The aim of the study was based on the World Health Organization (WHO) recommendations to implement this intervention in maternal healthcare service by maternal healthcare providers. To effectively implement this study and avoid methodological biases, health centers were selected. Primary health care services, particularly health centers, provide maternal healthcare to the majority of mothers. Again, during the cluster selection process, most hospitals have separate psychiatric outpatient clinics, and antenatal care is not served by the same clinicians (general practitioners, obstetricians, emergency surgeons, midwives/nurses, and health offices). Therefore, we selected health centers to balance the baseline cluster characteristics, directly access maternal healthcare providers, particularly midwives/nurses, and avoid related methodological biases.

In this study, participant identification and consent were obtained before the randomization process. The randomization of clusters was done by Statistical Package for the Social Sciences (SPSS)-generated random sequences into intervention and control arms at a 1:1 allocation ratio. A person, who was blinded to the study groups and did not participate in this study, randomized the clusters and allocated them into two groups. Similarly, the study was single blind. Only the outcome assessors were concealed, were not informed about the allocation and objective of the study, did not live in any of the clusters, and were not trial implementers.

### Outcome

The primary outcome variable was the PPD literacy score of a mother, which was compared between intervention and control groups. It comprises 31 items with the following seven categories: Ability to recognize PPD (six items), knowledge of risk factors and causes (five items), knowledge and belief of self-care activities (five items), knowledge about professional help available (two items), beliefs about professional help available (two items), attitudes that facilitate recognition of PPD and appropriate help seeking (six items), and knowledge of how to seek information related to PPD (five items) (6). Higher scores indicated higher literacy. Additionally, variables associated with the overall PPD literacy score of a mother were used as secondary outcome variables (**Supplementary File 2** description of study variable).

### Intervention description

The study used antenatal group-based psychoeducation interventions. The intervention content and psycho-educational techniques used were derived from different well-validated guidelines, including the WHO, “Guide for Integration of Perinatal Mental Health in Maternal and Child Health Services” and Clinical Practice Guidelines for Psychoeducation (17, 18, 26). The primary goal of psych-education is to provide the client with comparable types of problems and their families’ knowledge of the various aspects of the illness (in this case PPD), its prevention, and therapy. As a result, they can work with professionals and families to achieve a better overall outcome (17, 18). Furthermore, the intervention was given in groups, which offered its own set of benefits for the pregnant women. The majority of common questions raised by pregnant women were addressed predominantly through discussion and experience sharing within the groups, which helped the pregnant women feel like they were not alone and more confident in the group’s support (27, 28). It is flexible and easy to implement, and it also helps manage attendance barriers (17, 18). Furthermore, the WHO recommended it for busy, low- and middle-income country health facilities by non-specialized healthcare providers in non-specialized healthcare facilities (18, 19).

The treatment group received usual care plus five antenatal group-based psycho-education classes, while the control group received only usual care. The usual antenatal care includes screening, prevention, education, and counseling, as well as maternal and fetal assessments to prevent common pregnancy problems and complications. However, this care does not provide intervention explicitly aimed at maternal mental health services (15). Antenatal group-based psychoeducation was administered to mothers face-to-face in groups through discussion and experience sharing. In each group, up to 8–10 women participated. In total, five antenatal group sessions lasting 60 to 90 min were administered for each group for 5 weeks (17, 26). Generally, the study took approximately 8 months, from 28 March to 1 December 2022. The study participant was enrolled at 12–20 weeks of gestation, and end-line data were collected at 6 weeks postpartum, which included the following:

Session 1: Baby blue and post-partum depression. The session includes a discussion on baby blue and PPD, how it is seen in the community, stigma related to PPD, and their beliefs toward PPD, definition of PPD, the difference between baby blue and PPD, risk factors/causes of PPD, potential impact on the mother, pregnancy outcome, and long-term effect on the child’s health and family as a whole.
Session 2: Symptoms of PPD and when to recognize it. A discussion was conducted on the signs and symptoms of PPD, management, and treatment options.
Session 3: Prevention methods (self-care activities). A discussion was conducted on prevention methods based on common causes and risk factors that are common during pregnancy and postpartum such as social support. Additionally, the healthy and unhealthy ways of coping with stress (more self-care activities) were discussed in detail.
Session 4: Development of social support. A group discussion and experience sharing were held on the benefit of social support throughout pregnancy and after the baby is born, as well as its use in stress management, resources of social support, and how to receive them.
Session 5: Family member participation. Partners or family members (who were immediate caretakers for the mother) were invited, and discussions were held with them on various aspects of PPD and its definition, risk factors, potential impact on the mother, pregnancy outcome, and long-term effect on the baby and family as a whole. A comprehensive description of the intervention is provided in Ref (29). on pages 4–7. Additionally, **Supplementary File 3** training manual has the details.

### Data collection tools and producers

We employed a well-validated, pre-tested, and structured instrument to collect the data. The English Version of the PPD literacy scale (PoDLiS) was taken for this studies (6, 30). It is designed based on the definition of mental health literacy, qualitative studies, and preliminary screening of pregnant and postnatal women. It includes 31 items with seven categories and has been used for assessing the maternal PPD level of literacy. It was tested on perinatal women and demonstrated strong reliability and validity in this population. The overall original PoDLiS has a Cronbach’s α coefficient of 0.78, and the content validity index (CVI) ranges from 0.80 to 1. The construct validity was evidenced by a χ²/df of 1.38, a root mean square error of approximation (RMSEA) of 0.040, a standardized root mean square residual (SRMR) of 0.074, a comparative fit index (CFI) of 0.919, an incremental fit index (IFI) of 0.921, and a goodness-of-fit index (GFI) of 0.871 (6). Higher scores indicated higher literacy (6, 30).

For this specific study, principal component analysis (PCA) was done. Two items were removed from the subscale of knowledge on how to seek information related to PPD, which scored less than 0.5 points (information from the internet and radio). Finally, all the assumptions were checked and fitted. Bartlett’s test of sphericity was significant at p < 0.0001. The Kaiser–Meyer–Olkin (KMO) measure of sampling adequacy was >0.79, and the total variance explained was 72.2%. The reliability of items (inter-item consistency) was checked (Cronbach alpha value >0.83). Furthermore, to ensure consistency of meaning, language experts separately translated the English-adapted tool into two local languages, Oromo and Amharic.

Data collectors and supervisors received training. Eight Bachelor of Science (BSc) midwives and two Master of Science (MSc) in Psychiatry and Midwifery professionals, respectively, collected and supervised the data. For the pretest, 5% of pregnant women outside the study area underwent the test. Data collection occurred between 12 and 20 weeks of gestation and 6 weeks postpartum through face-to-face interviews. We immediately checked the data for completeness, quality, and clarity. We identified, communicated, deliberated, and resolved all encountered challenges before the next day for data collection details found in Ref (29) (p. 7).

### Data analysis

We used the Statistical Package for the Social Sciences (SPSS) version 25 to analyze the data. Rigorous preparation was undertaken before data collection to enhance data quality. Additionally, preprocessing was conducted through descriptive analysis to identify missing data, errors, and outliers and facilitate subsequent analysis. To check for missing data, the number of cases missing per variable, the number of variables missing per case, and the pattern of correlations among variables were checked. Based on this analysis, we identified a few missing data points. For those with a few numbers of missed data, imputation techniques were used by revising the original questionnaire and using the average value derived from the other variables’ information to maintain the integrity of the data. Descriptive statistics were presented as the frequency with a percentage or mean ± SD.

We checked all quantitative data for normality using statistical (Shapiro–Wilk test) and/or graphical (PP-plot and histogram) methods as appropriate. We used intention-to-treat analysis to include all enrolled pregnant women in the final analysis. We assessed the PPD literacy score of the mothers using a five-point Likert scale asking respondents to indicate on the questionnaire that their answer to each question was either 5 (strongly agree), 4 (agree), 3 (neutral), 2 (disagree), or 1 (strongly disagree). We also applied reverse coding to the negatively stated questions (6). Generally, we classify Likert 1–5 score mean ranges of 1–2.4, 2.5–3.4, and 3.5–5 as low, neutral, and high, respectively (31, 32). We classified the PPD literacy score into low, neutral, and high based on this classification. Furthermore, the multivariate models were adjusted for educational status and parity among the intervention and control groups controlling for factors that might affect the outcome through analysis of covariance (ANCOVA) and multiple analysis of covariance (MANCOVA) due to significant differences showed between the two groups on maternal educational attainment and parity.

Finally, we analyzed the odds ratio using a generalized linear mixed model (GLMM) taking into account the clustering effect with a 95% confidence interval and the p-value at a significance level of 5%. We checked the model fitness based on a small value of the Akaike information criterion (AIC). To determine statistical significance, a p-value <0.05 was employed. The adjusted odds ratio (AOR) and 95% confidence interval (CI) were used to demonstrate the strength of the association and level of significance. The target (dependent) variable in this study was the mean PPD literacy score. Based on a previously done study on PPD literacy, we first selected the variables (educational status, job, marital status, unplanned pregnancy, complication during labor, history of mental health, partner emotional support, coping mechanism, self-esteem, and antenatal group-based psychoeducation intervention) as independent variables, and initial analysis was done. We then incorporated the factors with a significant association from the primary model (marriage status, unplanned pregnancy, partner emotional support, coping, self-esteem, and antenatal group-based psychoeducation intervention) into the final model. Finally, we reposted the variables that had a significant association in the final model.

## Results

**Figure 1:**
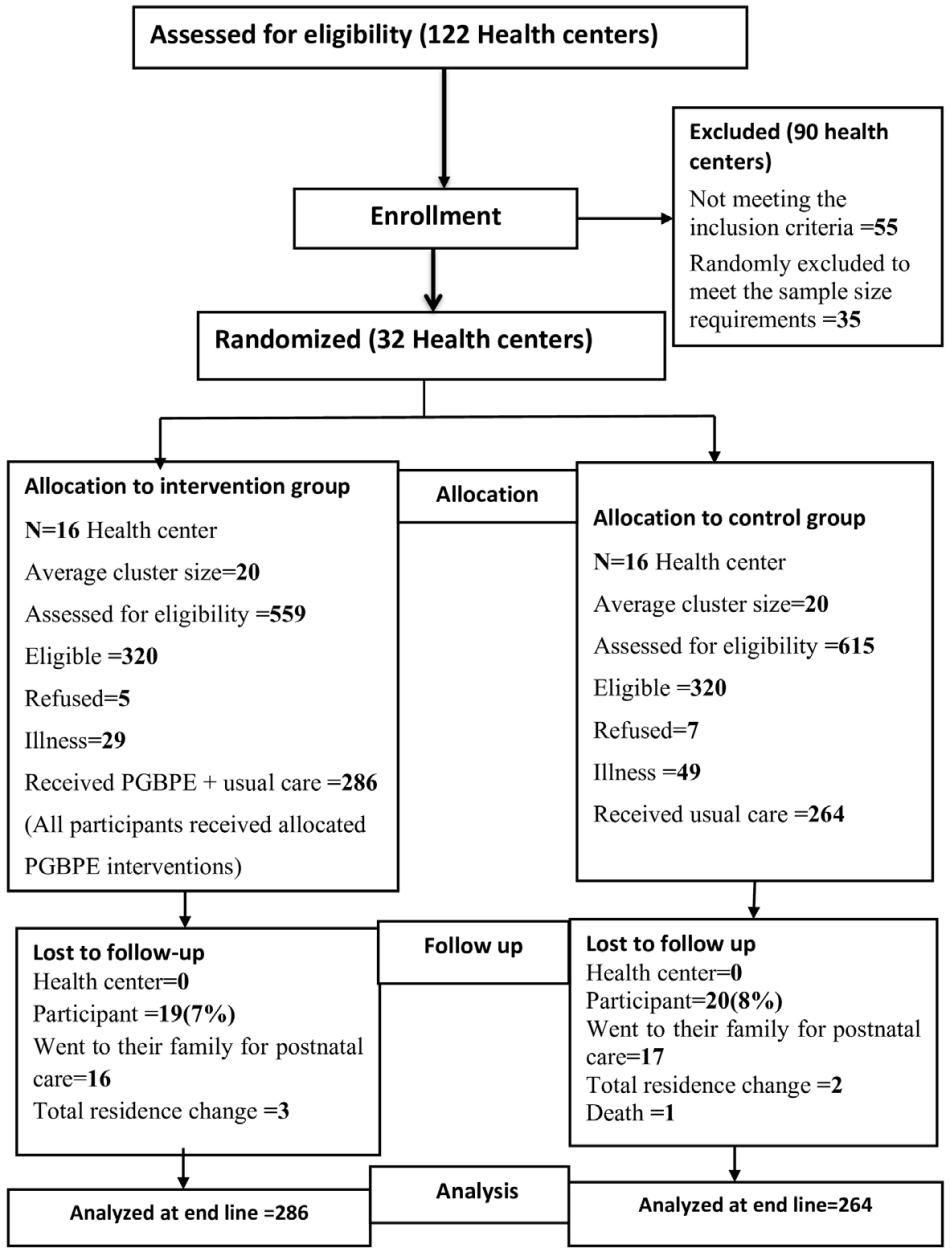
CONSORT 2010 trial flow chart, intervention study, Jimma Zone, 2023.

**Figure 1** shows the flow of the study. We randomized thirty-two (32) non-adjusted health centers (16 in each arm). Out of the 640 eligible women, only 550 gave their consent and were enrolled in the study (antenatal group-based psychoeducation intervention = 286 and routing antenatal care = 264). The study lost 19 intervention participants and 20 control participants due to postpartum care location changes and deaths. Finally, we collected end-line data from 511 participants, 267 (52.3%) from the intervention group and 244 (47.7%) from the control groups, resulting in an overall response rate of 92.9%. We used intention-to-treat analysis and included all enrolled participants (N = 550) in the final analysis (**Figure 1** Trial flow).

### Demographic variable

More than half of the participants live in the urban area: 328 (59.6%). Most of the participants were in marital unions, 484 (94.7%), and housewives, 521 (71.1%). The mean age of participants was 27.19 years old (SD ± 5.7) (intervention = 26.8 ± 5.8, control =27.7 ± 5.6). Two hundred sixteen participants (39.3%) have not attained formal education, whereas 204 (37.1%) have attained primary education. Furthermore, the majority of the participants, 344 (62.5%), were primipara, and only 24 (4.4%) had a history of mental health. We also compared the demographic variables between the intervention and control groups. There was no statistically significant difference between the two groups except for educational status and parity. To control the confounding effect of these two variables, the multivariate models were adjusted through analysis of covariance (ANCOVA) and multiple analysis of covariance (MANCOVA) (**Table 1**).

**Table 1:** Comparison of background variable (N = 550, IG = 286, CG = 264).

Variable with or without category	IG: N (%)/(mean ± SD)	CG: N (%)/(mean ± SD)	p-Value
Residence			
Urban	164 (57.3)	167 (63.3)^†^	0.15
Rural	122 (42.7)	97 (36.7)	
**Age**	26.8 ± 5.8	27.7 ± 5.6^††^	0.14
Marital status			
Married	272 (95.1)	249 (94.3) ^†^	0.54
In-relationship	10 (3.5)	9 (3.4)	
Others*	4 (1.5)	6 (2.4)	
Education status			
Informal education	**125 (43.7)**	**91 (34.5)** ^†^	**0.04**
Primary (1–8)	**104 (36.4)**	**100 (37.9)**	
Secondary (9–12)	**35 (12.2)**	**56 (21.2)**	
College/above	**22 (7.7)**	**17 (6.4)**	
Job			
Housewives	206 (72)	185 (70.1)^†^	0.67
Private works	42 (14.7)	44 (16.7)	
Government employees	27 (9.4)	22 (8.3)	
Domestic workers	11 (3.8)	13 (4.9)	
Monthly income/ETB (overall median = 3,500.0)	4,345.8 ± 3,124.9	4,521.0 ± 3,150.9^††^	0.26
Parity			
**Primipara**	**198 (69.2)**	**146 (53.3)** ^†^	**0.01**
**Multipara**	**88 (30.8)**	**118 (44.7)**	
Unplanned pregnancy for the mother			
Yes	65 (22.7)	58 (22)^†^	0.81
No	221 (77.3)	206 (78)	
History of mental health			
Yes	14 (4.9)	10 (3.8)^†^	0.56
No	272 (95.1)	254 (96.2)	
Social support			
Adequate	63 (22.03)	60 (22.73)^†^	0.84
Inadequate	223 (77.97)	204 (77.27)	
PPD literacy			
Literate	78 (27.27)	77 (29.17)^†^	0.62
Illiterate	208 (72.73)	187 (70.83)	

Others* = divorced, separated, widowed. IG, intervention group; CG, control group.

^†^χ² test.

^††^t test.

Bold values indicate P-value < 0.05.

### Post-partum depression literacy

Overall, the study revealed that antenatal group-based psycho-education intervention offered by maternal healthcare providers was effective in improving the PPD literacy score of postpartum mothers. Compared to controls, the total PPD literacy score was higher among the intervention clusters {[228 (79.7%) vs. 176 (66.7%)], p = 0.001}. Furthermore, the study demonstrated significant difference between groups regarding the overall mean PPD literacy score (intervention, 3.75 ± 0.46; control, 3.48 ± 0.46; ηp² = 0.07), the ability to recognize PPD (intervention, 4.30 ± 0.64; control, 3.94 ± -.75; ηp² = 0.06), knowledge of risk factors and causes of PPD (intervention, 4.03 ± 0.69; control, 3.67 ± 0.70; ηp² = 0.05), and access to PPD related information (intervention, 3.28 ± 1.25; control, 2.01 ± 1.13; ηp² = 0.21) at p = 0.001. Furthermore, the study showed a marginally significant result between groups regarding the subscale of knowledge and belief of self-care activities (intervention 4.37 ± 0.54, control 4.26 ± 0.50, ηp² = 0.01, p = 0.051). However, only marginally significant results were seen; both groups achieved the highest scores (>90%) (intervention = 268; 93.8%; control = 238; 90.1%). Conversely, there were no significant differences in knowledge of professional help available (intervention 2.97 ± 1.13, control 2.83 ± 0.80, ηp² = 0.00, p = 0.303), beliefs regarding professional help available (intervention 2.67 ± 0.89, control 2.50 ± 0.72, ηp² = 0.01, p = 0.063), and attitudes toward PPD recognition and appropriate help seeking (intervention 3.91 ± 1.02, control 3.91 ± 1.02, ηp² = 0.00, p = 0.586) (**Table 2**).

**Table 2:** Comparison of PPD literacy score and effect of size between groups (N 550, IG = 286, CG = 264).

Dimensions	Groups	Score (N/%)			Mean ( ± SD)	Effect of size/ partial eta squared	p-Value
		Low	Neutral	High			
Total PPD literacy score	CG	7/2.7	81/30.7	176/66.7	**3.48/0.46**	**0.07**	**0.001**
	IG	1/0.35	57/19.9	228/79.7	**3.75/0.46**		
Ability to recognize PPD	CG	7/2.7	59/22.3	198//75.0	**3.94/0.75**	**0.06**	**0.001**
	IG	1/0.3	29/10.2	256/89.5	**4.30/0.64**		
Knowledge of risk factors and causes	CG	14/5.3	78/29.5	172/65.2	**3.67/0.70**	**0.05**	**0.001**
	IG	7/2.4	53//18.5	226/79.0	**4.03/0.69**		
Knowledge and belief in self-care activities	CG	1/0.4	25/9.5	238/90.1	4.26/0.50	0.01	0.051
	IG	1/0.3	17/5.9	268/93.8	4.37/0.54		
Knowledge about professional help available	CG	85/32.2	128/48.5	51/19.3	2.83/0.80	0.00	0.303
	IG	94/32.9	81/28.3	111/38.8	2.97/1.13		
Beliefs about professional help available	CG	128/48.5	117/44.3	19/7.2	2.50/0.72	0.01	0.063
	IG	105/36.7	122/42.7	59/20.6	2.67/0.89		
Attitudes toward recognition of PPD and appropriate help seeking	CG	26/9.8	72/27.3	166/62.9	3.91/1.02	0.00	0.586
	IG	22/7.7	74/25.9	190/66.4	3.91/1.02		
Knowledge of how to seek information related to PPD	CG	183/69.3	42/15.9	39/14.8	**2.01/1.13**	**0.21**	**0.001**
	IG	61/21.3	46/16.1	179/62.6	**3.28/1.25**		

IG, intervention group; CG, control group.

Bold values indicate P-value < 0.001.

Furthermore, the multilevel result shows that mothers who received intervention (AOR = 0.23, 95% CI = 0.08–0.39), had emotional support from a partner (AOR = 0.1, 95% CI = 0.02–0.17), used unhealthy ways to deal with stress [AOR = −0.14, 95% CI = −0.22–(−9.07)], and multiparty [AOR = −0.15, 95% CI = −0.22–(−0.08)] were significantly associated with overall PPD literacy score.

## Discussion

This cluster-randomized controlled trial showed that relative to the control group, the antenatal group-based psychoeducation intervention resulted in significant improvement in the overall PPD literacy score among postnatal mothers. However, the intervention effect was different for each attribute of PPD literacy. Significant improvements were seen between arms only in the ability of PPD recognition, knowledge of risk factors and causes of PPD, and knowledge of how to seek information related to PPD. The result of this study is supported by different studies that revealed that educational interventions have improved the overall perinatal mental health and/or PPD literacy score of mothers, particularly on PPD recognition, knowledge of risk factors and causes of PPD, knowledge on how to seek information related to PPD, and knowledge and belief about self-care activities (5, 33–35).

This study also shows that subscales related to the knowledge and belief of self-care activities yielded marginally significant results for both the intervention and control groups. However, only marginally significant results were seen; both groups achieved the highest scores (>90%). This might be because traditional mothers, particularly those in low-income countries like Ethiopia, are culturally aware of perinatal mental health issues and most of the self-care activities that help prevent perinatal mental illness. Furthermore, most of the questions related to self-care activities can be answered by general knowledge related to mental health. However, there is little to no evidence showing that culturally prescribed care in traditional society protects mothers from PPD (36). Similarly, in this study, the statistical results showed that a high proportion of mothers in the control group had PPD compared to the intervention groups (29). Therefore, even though their scores are high, they should receive practical and professional assisted care.

Furthermore, the statistical analysis showed that this intervention did not affect subscales of knowledge about professional help available, beliefs about professional help available, and attitudes toward recognition of PPD and appropriate help seeking. Similarly, studies also revealed that perinatal women had low attitudes toward seeking help from formal sources (professionals) for perinatal mental health issues preferring instead to seek support from informal sources (family, friends, and social and religious help) due to various personal, cultural, and structural barriers (5, 33, 36, 37). Similarly, the multilevel result in this study strengthens this evidence. It indicates that mothers who received emotional support from their partners scored higher overall in PPD literacy score than mothers who had no emotional support. Furthermore, different studies showed that mothers with favorable partner emotional support had favorable mental health literacy and mental health status (36–39). Evidence also demonstrates that women’s perceptions of their male partners’ encouragement to seek professional help influenced their intention to do so (38, 39).

Furthermore, a mother’s strong perceptions of the social, religious, and cultural effects of PPD result in stigmatizing attitudes and beliefs toward professional help. Due to their strong beliefs on social and cultural relations of mental health, even with their awareness of mental health-related problems, they continue to regard professional and medical support as less significant for addressing PPD (33, 40, 41). Similarly, some studies show that even though they have access to health or social services, they give priority to family dignity and are sensitive to family secrets (42).

Furthermore, a systematic review study revealed beliefs that are a direct contradiction with literacy theory. It indicates that most women believe that PPD is not a problem and that their struggles are a normal part of motherhood that will go away on their own. Due to these perceptions, they are reluctant to learn and seek professional help (5, 43). Correspondingly, this study showed that mothers who used dysfunctional coping mechanisms had low overall PPD literacy scores. The reason may be that mothers who employed dysfunctional coping mechanisms mostly lacked knowledge about healthy stress management skills, which could also have a direct correlation with their PPD literacy level. Moreover, the easy unavailability of professionals, distance/lack of transportation, and time constraints related to childcare were mentioned by different studies (5). All these factors may push mothers to have poor attitudes toward having knowledge and beliefs related to professional help.

In general, this antenatal group psychoeducation intervention did not affect professional help-related components of PPD literacy. In addition to the aforementioned discussions, the reason may be related to the use of self-report methods of data collection in this study. It is effective for gathering data from large sample sizes, but it may have limitations like social desirability bias, memory biases, and erroneous responses. Additionally, a primary objective of this study was the prevention of PPD within maternal health care services; we recruited non-depressed pregnant women, and the intervention primarily emphasized social support and self-care activities that required the mother’s active participation to avert PPD. These may be some of the factors that decrease the effectiveness of this intervention on subscales related to the professional help available. However, evidence shows depressed mothers should get and be treated by professionals because, in the perinatal period, untreated depression has severe effects on mothers and children, as well as their families and societies in general (11). This study excluded depressed mothers and referred them for professional help. Therefore, further study is necessary to explore and improve mothers’ formal help-seeking attitudes, particularly for moderately to severely depressed mothers.

Moreover, based on the results of this study, we can clearly understand the areas of adequate and poor PPD literacy these mothers had. This aids in identifying the areas that require intervention. So, for future studies, rather than directly implementing psycho-education interventions, a digital tool that assesses mothers’ PPD literacy should be attached to the psychoeducation intervention package.

### Limitations

The study used self-report methods, it is effective for gathering data from large sample sizes, but it may have limitations like social desirability bias, memory biases, and erroneous responses. To avoid these, a validated instrument and well-trained data collectors were used. However, it constitutes a limitation of our study. Despite using the same population, we only compared the outcome variable between the intervention and control groups based on end-line data, as the experiences of pregnancy and postpartum periods varied. Moreover, the trial employed a single-blind design, blinding only the outcome assessors. Moreover, early pregnancy discomfort and the mother’s places of delivery were not exclusion criteria. Thus, this situation has caused a decrease in participation and an increase in the loss of follow-up (49/550 × 100 = 8.9%). However, we conducted an intention-to-treat analysis, and a loss to follow-up of less than 20% cannot cause a significant validity problem (44).

## Conclusions and recommendations

The study showed that antenatal group-based psychoeducation had a significant effect on the overall PPD literacy score of mothers in primary healthcare units in Ethiopia. However, the effectiveness of the intervention varied across different subscales of PPD literacy score. The intervention significantly enhanced the subscales measuring the ability to recognize PPD, the knowledge of risk factors and causes of PPD, and the ability to seek PPD-related information. This study also shows that subscales related to the knowledge and belief of self-care activities yielded marginally significant results for both the intervention and control groups. However, only marginally significant results were seen; both groups achieved the highest scores (>90%). However, the results indicated that the intervention had no impact on the subscales measuring knowledge about professional help available, beliefs about professional help available, and on the subscales measuring attitudes toward PPD recognition and appropriate help seeking. Furthermore, partner emotional support, dysfunctional coping, and multiparty were significantly associated with the PPD literacy score of postpartum mothers.

Therefore, primary healthcare institutions in Ethiopia should implement this intervention method to promote maternal literacy regarding PPD within maternal healthcare services. However, to effectively change the negative attitudes toward seeking professional help, further research on interventions is necessary. Additionally, this study highlighted mothers’ preferences for both formal and informal assistance in preventing PPD. Therefore, instead of directly implementing a psychoeducation intervention, it would be beneficial to include a digital tool that assesses mothers’ PoDLiS as part of the psychoeducation intervention package.

## Data Availability

The original contributions presented in the study are included in the article/**Supplementary Material**. Further inquiries can be directed to the corresponding author.
