# Supplementary material for: Antenatal group-based psychoeducation to improve postpartum depression literacy in primary health care institutions in Ethiopia: a cluster randomized controlled trial
Source: Front Psychiatry. 2025 Apr 17;16:1548356. doi: 10.3389/fpsyt.2025.1548356 (PMC12043592; doi:10.3389/fpsyt.2025.1548356)
Supplement: Supplementary file 1 [file DataSheet1.zip › Data Sheet 1/Supplementary file 2.pdf]

Table 1. Description of the study variables

Variable	Description	Measurement
**Dependent variable**		
Residence	The place where the respondent usually belongs	Coded as 'urban' and 'rural'
Age	Age of the mother in completed years	A continuous variable and recoded into three categories: 15–24, 25–34, and ≥35
Education	The highest level of education the mother completed	Categorized into four groups: informal education, primary education (1–8), secondary education (9–12), and college and above
Marital status	Marital status of the mother	Categorized into three categories: married, in-relationship, and other (divorced, widowed, separated)
Job	The job of the mother	Categorized into four groups: housewife, private worker, government employee, and domestic worker
Estimated household income	The average monthly household earnings	A continuous variable and recoded into three categories: <3000, 3001–5000, and >5000
Parity	The number of times that a mother has been pregnant	Coded as 'Primipara' or 'Multipara'
Unwanted pregnancy (mother)	Mothers were asked if the pregnancy occurred when no children or no more children were desired (on her side)	Coded as 'Wanted' or 'Unwanted'
Complication during labor	The mother was asked if she faced complications during labor (like prolonged labor, obstructed labor, or operative delivery)	Coded as 'Yes' or 'No'
Literacy	We assessed the Postpartum Depression Literacy score (PoDLiS) of the mothers using a five-point Likert scale, asking respondents to indicate on the questionnaire that their answer to each question was either 5 (strongly agree), 4 (agree), 3 (neutral), 2 (disagree), or 1 (strongly disagree). We also applied reverse coding to the negatively stated questions [22].	Generally, we classify Likert 1-5 score mean ranges of 1-2.4, 2.5-3.4, and 3.5-5 as low, neutral, and high, respectively[23, 24]. Moreover, the higher score indicates the higher PPD literacy.
History of mental illness	History of mental illness (e.g., eating disorder, psychosis, bipolar disorder, schizophrenia)	Coded as 'Yes' or 'No'
Postpartum	Mothers were assessed by the PHQ-9	Score: (0–9) normal, (≥10)

depression	tool during the 6-week postpartum period. It comprises 9 questions, each having scores of 0–3, with minimum and maximum scores of 0 and 27, respectively.	depressed (52-54).
Self-esteem	Measured by the 10-item scale with a 4-point scale, ranging from 1 point (strongly disagree) to 4 points (strongly agree)	A continuous variable and computed. The mean for overall self-esteem was computed and mothers who scored above the mean were considered to have good self-esteem and those who computed below the mean were considered as poor self-esteem (70).
Coping strategy	Coping skill comprises 28 items with three dimensions of coping strategy: problem-focused, emotion-focused, and avoidance or dysfunctional coping.	The mean of the three dimensions of coping strategy was computed and mothers who scored at and above the mean were considered as having good coping skills, and those who computed below the mean were considered as having poor coping skills (57)
Interventions	Health centers/clusters were identified as intervention and control based on the intervention (group based on psycho-education) received	Coded as intervention = '1' and control = '0'
Social support	A functional social support questionnaire was used for the measurement of social support. It has 14 items with four categories: quantity of support, confidant support, affective support, and instrumental support.	After tool validation, the ordinal-rated response options were replaced with dichotomous (i.e., yes/no) variables. So, it was recoded as (yes = '1', no = '0'). In the end, the mean for overall social support was computed and mothers who scored above the mean were considered to have adequate social support and those who computed below the mean were considered to have inadequate social support (55)
