# Supplementary material for: Antenatal group-based psychoeducation to improve postpartum depression literacy in primary health care institutions in Ethiopia: a cluster randomized controlled trial
Source: Front Psychiatry. 2025 Apr 17;16:1548356. doi: 10.3389/fpsyt.2025.1548356 (PMC12043592; doi:10.3389/fpsyt.2025.1548356)
Supplement: Supplementary file 1 [file DataSheet1.zip › Data Sheet 1/Supplementary File 3.pdf]

Prenatal Group-Based Psycho-education for pregnant women

Having And Raising A Healthy Baby Is Hard Work.

Jimma University Institute of Health

Faculty of Health Science School of Public Health

Department of Population and Family Health

September, 2021

Jimma University

Acknowledgement

This prenatal group-based psych education Manual has been prepared for primary health care provider in preventing postnatal depressions. We would like to acknowledge the contributors of Prof. Zewdie Birhanu(Health, Behavior and Society), Dr. Muluemebet Abera (Associate professor of reproductive health), Dr.Mathios Mesaane (Psychiatrist),Yonas Tesfaye(Assistant Prof., in Psychiatry Nurse), and Bonsa(Assistant Prof., psychologist), Biru abdisa(Assistant Prof. and Marta Tessema(MSc, in clinical Midwifery,PhD.Fellow) from Jimma University.

Table of content

Contents

Acknowledgement 2
Table of content 2
Notes to the trainer 3
  Methodology 3
Welcome, expectations, objectives 3
Materials and preparation 3
Session one:-Baby blue and postpartum depression 4
Session two: Symptoms of PPD and when to recognize it 7
Session three: - Prevention methods 9
Session four: Social Support as prevention methods 16
Session five: invitation and discussion with family 21
Annexes 22
  Provider observation checklist 22
  Prenatal group based spycho-education reporting format 22
  Reference 23

Notes to the trainer

Methodology

Conduct the entire training session in a circle to reinforce the atmosphere of respect, attention, trust, sincerity, and empathy that characterizes a group. New knowledge, attitudes, and skills should be discussed by health care provider. Use the log frame during each of the training sessions to pinpoint the objectives and activities. The objectives of the project, activities (number of sessions, content covered in each session), and attendance for each participant mother (number of mothers who participated in each session and finished each session correctly) should be pointed out. Furthermore, interviews should be held with participants about their satisfaction and feedback on the intervention every day.

Welcome, expectations, objectives

Materials and preparation

Flip chart, note cards, markers, pen. Flip chart with learning objectives (covered until presented at the end of the session)

Time: 20 minutes

Activity

1. Open the session and welcome participants. Review the training schedule.

2. Divide participants into pairs. Ask each pair to introduce themselves and agree on one expectation they share for the workshop. After 3–5 minutes, ask each pair to introduce each other to the larger group and share the expectation. Facilitators should write each expectation on a flip chart sheet.

3. Compare participant expectations to training objectives.

At the end of the training the participant will be able to:-

Describe postpartum depression, its risk factors, symptoms, and consequences for the mother child and family as a whole.

Differentiate postpartum depression and baby blue

Describe healthy coping and prevent postpartum depression

Describe social support mean, its benefit for prevention of postpartum depression

Get social support for this perinatal period

Take action to prevent postpartum depression

Session one:-Baby blue and postpartum depression

Objectives

- Describe perinatal depression/mental health during postpartum period
- Discuss how its seen in our community ,beliefs, stigma behind PPD

Materials and preparation

- Flip chart
- Markers

Time 60-90 minute

Activity /Discussion:

1. Ask: What is perinatal mental illness/depression?
2. Listen to their responses and then share the following:

Baby Blues are common: Up to 80% of women experience Baby Blues. Baby Blues usually occur 2-5 days after delivery. With time, patient, and support, they go away.

About 1 in 5 women have PPD after they have a baby. PPD will require support to help you feel better. The chart below distinguishes between symptoms that are less serious (Baby Blues) and those that require support (PPD).

SYMPTOMS THAT ARE NOT SERIOUS	SYMPTOMS THAT REQUIRE SUPPORT
Several days of baby blues	Feeling very down for more than two weeks
Occasional worries that come and go	Relentless anxiety that never goes away
Negative feelings and thoughts that come and go	Negative feelings that outweigh the positive feelings
You can take care of yourself and your baby	You are unable to cope with your life or your baby
Some escape fantasies	Thoughts about harming yourself or your baby
Poor sleep due to caring for your baby	Not being able to sleep when your baby sleeps at night or needing to stay in bed all the time
Fatigue	Extreme exhaustion or agitation
Normal appetite with normal fluctuations, i.e., your appetite may increase if you are nursing	Compulsive overeating or ongoing loss of appetite
Some forgetfulness	Severe inability to concentrate and focus
Moments of sadness	Intense feelings of sadness that do not go away
Needing a break from your responsibilities and your baby	Avoiding your baby
Wanting to limit visitors and activities	Withdrawing from the world and becoming isolated
Occasional irritability and anger	Feelings of intense anger and irritability

3. Group discussion :
    - How it's seen in our community, beliefs, and stigma behind PPD?
4. Ask: what are the risk factors for PPD
5. Listen to their responses and then share the following:

<table>
<tr><th colspan="2">GENERAL RISK FACTORS FOR POSTPARTUM DEPRESSION</th></tr>
<tr><th>During pregnancy</th><th>During post-partum period</th></tr>
<tr><td>

Adolescent pregnancy, Unwanted pregnancy, Being unmarried or separated
Marital relationship: unsupportive; polygamous
Lack of practical support
Spouse/domestic violence
Previous stillbirth or repeated miscarriage, Null parity
Poverty and lack of financial resources
Pregnancy as a result of rape
Difficult relationship with in-laws
Antenatal depression or severe anxiety
Illnesses in this pregnancy, antenatal hospital admission, Past psychiatric history

</td><td>

Adolescent pregnancy, Unintended pregnancy, Unmarried
Difficulties with husband's behavior (physical violence; verbal abuse; alcohol use; being illiterate and unemployed; providing little assistance; rejecting the pregnancy)
Inability to confide in partner, Poverty (low income; lack of personal income generating activity; inadequate housing)
Overcrowding and lack of privacy, Antenatal depression or severe anxiety
Illnesses during pregnancy, antenatal hospital admission, operative birth
Past psychiatric history, Large number of children, Infant unsettled, sick, not thriving
Problematic relationship with in-law family (mother-in-law and sister-in-law)
Birth of a girl child in cultures over-valuing boy child
Lack of sustained, dedicated, practical care after birth for the culturally prescribed period and Other stressful life events

</td></tr>
<tr><td colspan="2">The most common are: - postpartum depression might be caused by stressful circumstances in the life (such as the death of a loved one or divorce, lack of social support such as intimate partner support and a previous history of depression.</td></tr>
</table>

6. What is the impact of PPD?
7. Listen to their responses and then share the following:

IMPACT ON THE MOTHER

Functional Impairment: - Depression affects women decision-making abilities and causes feeling of overly anxious, doubting her ability to care of her family. Not responding babies need appropriately and See baby's behavior is difficult, difficulty of exclusive breast feeding. Fail to recognize babies' cues that have potential impact on the development of the baby

Relationship Impairment: - irritability/ Feeling unusually sad and teary may be a symptom of postpartum depression, withdrawing from loved ones, feeling distant from her baby and thinking about hurting herself or her baby. This abnormal behavior and irritability of the women affects her interpersonal relationship and results couple dysfunctions, intimate partner violence and child abuse.

Clarity of Thinking: - it affects person's memory and concentration. So, mothers show maladaptive behaviors and disability in the areas of clarity of thinking, low self-esteem, poor cooping ability to any stress and lack of interest for everything. Negative view of motherhood

Self-Care: -Depression also affects a woman healthy life styles cause's loss of appetite and poor nutrition, lack of sleep, lack of adequate rest. Low health responsibility for herself and baby. Not following pregnancy care, it's linked to higher rates of spontaneous abortion, prolonged labour and operative deliveries. Risk of Suicide, substance abuse, risk of depression for next pregnancy

Session two: Symptoms of PPD and when to recognize it

Objectives

- Describe symptom of perinatal depression
- Discuss when to recognize PPD and seek help
- Describe prevention methods

Materials and preparation

- Flip chart

- Markers

Time 60-90 minute

Activity /Discussion

1. Recap. Ask: about symptoms of perinatal depression and its difference with baby blue
2. Listen to their responses and then share session one activity(1&2)
3. Ask: which of the symptoms they feel now?
4. Give 5 minute for the women to think about their feeling. And discuss again detail about the symptoms, duration, and severity to clearly separate the women.
5. Ask: is PPD preventable?
6. Listen to their responses and then share the following:

Yes PPD can be prevented mostly by the following action!!!!!

- Lean on Family and Friends
    - A few hours of weekly childcare can give you a much-needed break
    - Get help cleaning the house or running errands
    - Sharing feelings openly allows family & friends to provide support
- Focus on Wellness
    - Food: Eat breakfast, choose healthy snacks, avoid alcohol use
    - Exercise: Invite friends to go on walks, try a new activity
    - Self-care activities: Relax-do something nice for yourself, even if you are tired, doing something positive can improve your mood, take adequate sleep, rest, personal hygiene
- Find a Support Group
    - Other women in your community may suffer from postpartum depression
    - Sharing your feelings with a group of women experiencing the same thing can be helpful
    - Talk to your health care provider about how to join a group
- Talk to Health Care Professional
    - Professionals will know what options are available to you for help
    - Do not give up

- Look for a different provider if you feel your current provider does not understand what you are going through

➢ Seek Counseling From an Expert

- Many behavioral and maternal health specialists have training in postpartum depression
- They can give you a safe place to express your feelings and will be useful in helping you manage your symptoms

➢ Take Medication as Recommended by Your Doctor

- Talk to your health care provider about which medication, if any, is best for you
- Ask you're your health care about alternatives to medication

Session three: - Prevention methods

Objectives

- Describe prevention methods of PPD

Materials and preparation

• Flip chart

• Markers

Time 60-90 minute

Activity /Discussion

1. Ask: Prevention methods and what is done in our community to prevent PPD?
2. Listen to their responses and then share session two activity (5&6).
3. Ask: what is stress, and their experience(stress full situation women experienced before)
4. Listen to their response and then share the following

Types of Stress

Following are descriptions of the three types of stress

Positive stress results from adverse experiences that are **short-lived**.

Mothers may encounter positive stress when they attend a health facility, labor. This type of stress causes minor physiological changes including an increase in heart rate and changes in hormone levels.

With the support of caring partner/ friend or health care provider, mother can learn how to manage and overcome positive stress. This type of stress is considered normal and coping with it is an important part of the development process.

Brain storming

What are positive stresses prevalent during pregnancy and post-partum period?

Why you consider them as Positive?

b. Tolerable stress refers to adverse experiences that are **more intense but still relatively short-lived.**

Examples include the **death of a loved one**, **a natural disaster**, **a frightening accident**, and **family disruptions such as separation or divorce**.

If a mother has the support of friends, health care providers or responsible organizations, tolerable stress can usually be overcome. In many cases, tolerable stress can become positive stress.

N;B However, if the mother lacks adequate support, tolerable stress can become toxic and lead to long-term negative bio psychosocial effects.

Brainstorming:

- What are tolerable stresses prevalent with your client?
- Why you consider them as tolerable?

c. Toxic stress results from intense adverse experiences that may be sustained **over a long period of time—weeks, months or even years.** This type of stress can disrupt the **cognitive potential, compromise the functioning of important biological systems, and lead to long-term health problems.**

An example of toxic stress includes:

- **Abuse (intimate partner violence):** - Emotional, Physical, Sexual

Household Dysfunction: - Economic problem, husband cheating, Household mental

3.2 Coping

Since everyone has a unique response to stress, there is no "one size fits all" solution to managing it. No single method works for everyone or in every situation, so experiment with different techniques and strategies is the better way for designing an effective coping mechanism. Generally coping is effective when it focuses on what makes you feel stressed and what makes you feel calm and in control.

5. Ask: What is coping? How you acted in response to stress? And what you did to make yourself feel better?
6. Listen to their responses and then share the following scenarios:

Scenario 1:

W/r, Jamila is 7 months pregnant women. While her gestational age increases, she experienced tension having due lack of relative who will be with her at health facility during labor. After a while she asked her old friend to be with her when she go to hospital for delivery. Finally, her old friend become volunteer and gave birth a healthy baby at hospital.

Scenario 2: W/r Nesra is 7 months pregnant women. While her gestational age increases, she experienced tension having due lack of relative who will be with her at health facility during labor. So, she made a decision to not to ask anybody for help and give birth at home. Unfortunately, during labor, she bleeds much and she lost her child.

7. Ask: What kind of coping method was used these women? And what kind of coping methods would you use if you were in similar position?
8. Listen to their responses and then share the following:

Coping is the process by which people try to manage the perceived discrepancy between the demands and resources they appraise in a stressful situation. Since people engage in coping in an effort to neutralize or reduce stress, coping activities are geared toward decreasing the person's appraisal of or concern for this discrepancy.

Coping can serve two main functions.

a. It can alter the problem causing the stress (**Problem-focused coping)**

b. It can regulate the emotional response to the problem.(**Emotion-focused coping**)

In addition to the above classification from the point of an individual well-being, coping mechanisms can be divided in to two:

1. Healthy and
2. Unhealthy way of coping with stress

9. Ask: what do you think on healthy and unhealthy ways of coping? Share their negative experience?
10. Listen to their responses and then share the following:

Unhealthy ways of coping: it may temporarily reduce stress, but they cause more damage in the long run:

Taking out your stress on others (lashing out, angry outbursts, physical violence)<br><br>Overeating or under eating<br><br>Withdrawing from friends, family, and activities<br><br>Drinking too much or smoking	Using pills or drugs to relax<br><br>Sleeping too much<br><br>Procrastinating(postponing)<br><br>Filling up every minute of the day to avoid facing problems

Healthy ways of coping with stress Dealing with Stressful Situations: The Four A’s

**Change the situation:**	**Change your reaction:**
Avoid the stressor	Adapt to the stressor
Alter the stressor	Accept the stressor

Avoid unnecessary stress

Not all stress can be avoided, and it's not healthy to avoid a situation that needs to be addressed. You may be surprised, however, by the number of stressors in your life that you can eliminate.

Learn how to say "no" – Know your limits and stick to them. Whether in your personal or professional life, refuse to accept added responsibilities when you're close to reaching them. Taking on more than you can handle is a surefire recipe for stress.

Avoid people who stress you out – If someone consistently causes stress in your life and you can't turn the relationship around, limit the amount of time you spend with that person or end the relationship entirely.

Take control of your environment –avoid places that may causes you feel stress.

Avoid hot-button topics – If you get upset over competitions about house hold economy, marriage or work, cross them off your conversation list. If you repeatedly argue about the same subject with the same people, stop bringing it up or excuse yourself when it's the topic of discussion.

Pare down your to-do list – Analyze your schedule, responsibilities, and daily tasks. If you've got too much on your plate, distinguish between the "should" and the "musts." Drop tasks that aren't truly necessary to the bottom of the list or eliminate them entirely.

Alter the situation

If you can't avoid a stressful situation, try to alter it. Figure out what you can do to change things so the problem doesn't present itself in the future. Often, this involves changing the way you communicate and operate in your daily life.

Express your feelings instead of bottling them up: If something or someone is bothering you, communicate your concerns in an open and respectful way. If you don't voice your feelings, resentment will build and the situation will likely remain the same.

Be willing to compromise: When you ask someone to change their behavior, be willing to do the same. If you both are willing to bend at least a little, you'll have a good chance of finding a happy middle ground.

Be more assertive: Don't take a backseat in your own life. Deal with problems head on, doing your best to anticipate and prevent them. If you've got an exam to study for and your chatty roommate just got home, say up front that it is not the time to talk.

Manage your time better: Poor time management can cause a lot of stress. When you're stretched too thin and running behind, it's hard to stay calm and focused. But if you plan ahead and make sure you don't overextend yourself; you can alter the amount of stress you're under.

Adapt to the stressor

If you can't change the stressor, change yourself. You can adapt to stressful situations and regain your sense of control by changing your expectations and attitude.

Reframe problems: Try to view stressful situations from a more positive perspective. Rather than fuming about a traffic jam, look at it as an opportunity to pause and regroup, listen to your favorite radio station, or enjoy some alone time.

Look at the big picture: Take perspective of the stressful situation. Ask yourself how important it will be in the long run. Will it matter in a month? A year? Is it really worth getting upset over? If the answer is no, focus your time and energy elsewhere.

Adjust your standards: Perfectionism is a major source of avoidable stress. Stop setting yourself up for failure by demanding perfection. Set reasonable standards for yourself and others, and learn to be okay with "good enough."

Focus on the positive: When stress is getting you down, take a moment to reflect on all the things you appreciate in your life, including your own positive qualities and gifts. This simple strategy can help you keep things in perspective.

Adjusting Your Attitude: How you think can have a profound effect on your emotional and physical well-being. Each time you think a negative thought about yourself, your body reacts as if it were in the throes of a tension-filled situation. If you see good things about yourself, you are

more likely to feel good; the reverse is also true. Eliminate words such as "always," "never," "should," and "must." These are telltale marks of self-defeating thoughts.

Accept the things you can’t change

Some sources of stress are unavoidable. You can’t prevent or change stressors such as the death of a loved one, a serious illness, or a national recession. In such cases, the best way to cope with stress is to accept things as they are. Acceptance may be difficult, but in the long run, it’s easier than railing against a situation you can’t change.

Don’t try to control the uncontrollable. Many things in life are beyond our control—particularly the behavior of other people. Rather than stressing out over them, focus on the things you can control such as the way you choose to react to problems.

Look for the upside. As the saying goes, “What doesn’t kill us makes us stronger.” When facing major challenges, try to look at them as opportunities for personal growth. If your own poor choices contributed to a stressful situation, reflect on them and learn from your mistakes.

Share your feelings. Talk to a trusted friend or make an appointment with a therapist. Expressing what you’re going through can be very cathartic, even if there’s nothing you can do to alter the stressful situation.

Learn to forgive. Accept the fact that we live in an imperfect world and that people make mistakes. Let go of anger and resentments. Free yourself from negative energy by forgiving and moving on.

Make time for fun and relaxation

Beyond a take-charge approach and a positive attitude, you can reduce stress in your life by nurturing yourself. If you regularly make time for fun and relaxation, you’ll be in a better place to handle life’s stressors when they inevitably come.

Healthy ways to relax and recharge

- Go for a walk.
- Savor a warm cup of coffee or tea.

• Spend time in nature.	• Get a massage.
• Consult a trusted friend.	• Curl up with a good book.
• Sweat out tension with a good workout.	• Listen to music.
• Take a long bath.	• Watch a comedy

Session four: Social Support as prevention methods

Objectives

- Discuss social support
- Get social support for this pregnancy and child birth

Materials and preparation

• Flip chart

• Markers

Time 60-90 minute

Activity /Discussion

1. Ask: What does the word "support" mean to you?

2. Listen to their responses and then share the following:

Feeling support usually means that we feel a sense of trust, acceptance, self-worth, value, and respect. When we are supported we can share information better, learn new skills, talk about our thoughts and feelings, and feel connected to others.

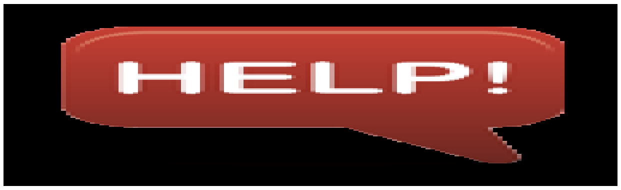

3. Ask the importance of social support during pregnancy and once the baby is born and its benefits in managing stress,

4. Listen to their responses and discuss with them

5. Ask forms of social support

6. Listen to their responses and then share the following:

Forms of Social Support

Support can come in many different forms:

- **Emotional Support:** This is what people most often think of when they talk about social support. People are emotionally supportive when they tell us that they care about us and think well of us.
    - ✓ **For example: -** if you separated from your partner or lost your job, a close friend might call every day for the first few weeks afterwards just to see how you are doing and to let you know that he or she cares.
    - ✓ Visits with friends and relatives
    - ✓ Or care what happens to you
    - ✓ Or give you love and affection
    - ✓ Or praise you for a good job
    - ✓ Or get calls from people you know
    - ✓ Give time Chances to talk to someone about problems at work or with my housework
    - ✓ Having Chances to talk to someone I trust about my personal and family problems
- **Practical Help:** People who care about us give us practical help such as
    - ✓ gifts of money or Chances to talk about money matters
    - ✓ Help when you need transportation
    - ✓ Help when you are sick in bed or gave birth
    - ✓ Help with money in an emergency /During child birth /post-partum
    - ✓ Help around the house, (house hold chore/ child care), assistance with cooking, child care, or help moving house.

    This kind of support helps us complete the basic tasks of day-to-day life.

- **Sharing Points of View:** Another way for people to help is to offer their opinion about how they view a particular situation, or how they would choose to handle it. In sharing points of view, we can develop a better understanding of our situation and the best way to handle it.

  For example, -

  - ✓ if you tell a friend about difficulties you are having with your mother in-laws or
  - ✓ Useful advice about important things in life e.g. marriage

She may offer a point of view you hadn't considered, and this may help you to better address the situation with your child.

- **Sharing Information:** It can be very helpful when family, friends or even experts give us factual information about a particular stressful event. For example, a friend who recently give birth might provide information about place birth, or a mother gave birth by operation might provide information about what to expect from different types of operations.

Getting Your Support Needs Met

Many of the people who are a part of our lives can provide social support. These can include our parents, spouse or partner, children, siblings, other family members, friends, co-workers, neighbors, health professionals and sometimes even strangers. We are unlikely to have all of our support needs met by just one person. Also, different people may be able to provide different types of support (e.g., our mother may be great at helping with child care, and our best friend might give great advice).

In general, the best support comes from the people we are closest to. Receiving support from people we have close emotional ties to does more for our emotional and physical health than support provided by people we are not particularly close to. For example, having close friends listen and care for you during a stressful time will likely do more for you than receiving the same support from someone whom you don't know very well.

7. Ask what is the advantage and disadvantage of social support?

8. Listen to their responses and then share the following

The advantage and disadvantage of social Support

Advantage

Expert recommends us that it is important to have at least one close friend. Having many close friends may not do us any better than having only two or three close friends. Social support does the best job of protecting us from the effects of stress when we believe that emotional support is easy to come by, and we have at least one person we can confide in.

Disadvantage

On the other hand, unhappy or poor-quality relationships with other people have been shown to have a negative impact on mental health and well-being. Conflictual, distressing relationships may do us more psychological harm than positive social relationships can do us good. The greatest benefit of social support may come from the protection from unhealthy relationships that it provides.

9. Ask how you can improve social support network

10. Listen to their responses and then share the following

How Do we Improve My Social Support Network?

1. **Reactivate social networks**» Identify the person's prior social activities that, if reinitiated, would have the potential for providing direct or indirect psychosocial support (e.g. family gatherings, outings with friends, visiting neighbors, social activities at work sites, sports, community activities). » Build on the person's strengths and abilities and actively encourage to resume prior social activities as far as is possible.
2. **Don't be afraid to take social risks:** Making new friends and acquaintances means that you will have to take some risks. You will need to seek out and introduce yourself to new people. Ask a friend to help you if this is hard for you (e.g., come to a hospital or clinic with you). If you have always been shy, and this has got in the way of you finding the

support you need, consider gaining the help of a professional. Informal gatherings/like edir, community centres/religious place, mother support groups, volunteer positions, schools and workplaces are also common places to meet people.

3. **Get more from the support you have:** While being careful not to overpower support providers, ask for what you need from others and be as specific as possible in your requests. It's a mistake to think that people will automatically know what you need - you will have to tell them.
4. **Ask for help:** Ask the people you know to help you broaden the network you have. If you have recently become single, ask your friends to introduce you to other single people your age. If you have recently come out, ask people you know who have gay friends to introduce you to them.
5. **Make a plan:** Figure out what kind of new support you need and brainstorm different ways you might be able to find it. Link to problem solving module once completed
6. **Create new opportunities:** Attend a religious activity and others. To meet new people, you will have to step outside your usual activities and lifestyle. If you just keep on doing what you always do, hoping to meet new people, you probably will fail.
7. **Let go of unhealthy ties:** This can be very difficult. Walking away from any relationship is painful. This is no less true because the relationship is doing you harming. But sometimes this is what we need to do. If all of your friends are involved in activities you want to avoid (e.g., spending money for no reason), you will need to let go of these friendships or risk getting back into your old habits. Use your judgments - sometimes we can simply spend less time with certain people (e.g., friends who don't like to exercise or meet new people) without abandoning the friendship altogether.
8. **Protect your marriage:** We know that a good marriage offers protection from depression and a bad marriage makes us vulnerable. If your marriage is faltering, do what you can to improve it. Don't be afraid to seek professional help when you need it.
9. **Be a joiner:** Sometimes the best way to find the support you need is through a support group. If you need support for a highly specific problem (e.g., raising a child with Downs/autism) you may only be able to find this support through a formal group setting.
10. **Be patient:** While very much worth the effort, making new friends is time-consuming. Recognize that you may need to meet many new people in order to make just one new

friend. Building intimacy also takes time. It may be several months from the time that you meet someone before you feel really close to them and that you can count on their support.

11. **Avoid negative relationships:** We know that negative, conflictual relationships are hard on our emotional health. The negative aspects may be obvious (e.g., abuse) but other times they can be more subtle (e.g., excessive dependence or over-controlling). As much as possible, avoid long-term relationships that are more negative than positive. Sometimes this can be hard - especially when these relationships are with family members. In this case, try to limit the amount of contact with these people (or buffer that contact with other helpful supporters), and avoid relying on them for support.

11. Ask: How to involve partners in child care and household chores positively

12. Make a group discussion and experience sharing

Session five: invitation and discussion with family

Objectives

- Describe the effect perinatal depression
- Give the social support need for the mother

Materials and preparation

• Flip chart

• Markers

Time 60-90 minute

Activity /Discussion

1. Ask: What does the word "PPD and its cause, effect on pregnancy, child, mother, partner, and family" mean to you?

2. Listen to their responses and then discuss section 1 and 2 in short:

3. Ask the symptom of PPD

4. Listen to their responses and then discuss the symptom in short (Table 1):

5. Ask area of help women wants from family/partner

6. Listen to their responses and then discuss forms of social support and its benefit for the women, child, and family/partner.

Annexes

Provider observation checklist

Healthcenter:_____________________________Date:______________Time:______________________sessi

on:_______________________Provider(s):_______________________________________

1 The facilitator(s) introduce themselves to the group.

1 The facilitator(s) clearly explain the day's session.

1 The facilitator(s) ask questions that generate participation.

1 The facilitator(s) motivate the quiet women to participate.

1 The facilitator(s) apply communication skills.

1 The facilitator(s) adequately manage content.

1 Women share their own experiences.

1 The participants sit in a circle.

1 The facilitator(s) thank the women for participating.

1 The facilitator(s) ask women to recap for next time to a pregnant woman, recap what they have learned, on last session.

Prenatal group based spycho-education reporting format

Facilitator's name:	Health center:	
Training data	Topic	Number of participants

Challenges: ---

Questions:--

Successes: --

Reference

O'Hara, M.W. and K.L. Wisner, *Perinatal mental illness: definition, description and aetiology.* Best Pract Res Clin Obstet Gynaecol, 2014. **28**(1): p. 3-12.

Fekadu Dadi, A., E.R. Miller, and L. Mwanri, *Antenatal depression and its association with adverse birth outcomes in low and middle-income countries: Step 4: Review practice against evidence-based audit criteria.* PLoS One, 2020. **15**(1): p. e0227323.

Ongeri, Lukens EP, Mcfarlane WR. Psychoeducation As Evidence-Based Practice: Considerations For Practice, Research, And Policy. Brief Treatment And Crisis Intervention. 2004;4(3):205-

Desta M, Memiah P, Kassie B, Ketema DB, Amha H, Getaneh T, Et Al. Postpartum Depression And Its Association With Intimate Partner Violence And Inadequate Social Support In Ethiopia: A Systematic Review And Meta-Analysis. Journal Of Affective Disorders. 2021;279:737-48. 45

National Institute Of Health And Care Excellence(192). Antenatal-And-Postnatal-Mental-Health-Clinical-Management-And-Service Guidance(2014).Wwwniceorguk/Guidance/Cg192.{Accessed December 17.2014}.

Johnson JE, Wiltsey-Stirman S, Sikorskii A, Miller T, King A, Blume JL, Et Al. Protocol For The ROSE Sustainment (ROSES) Study, A Sequential Multiple Assignment Randomized Trial To Determine The Minimum Necessary Intervention To Maintain A Postpartum Depression Prevention Program In Prenatal Clinics Serving Low-Income Women. Implement Sci. 2018;13(1):115.
