# Supplementary material for: Antenatal group-based psychoeducation to improve postpartum depression literacy in primary health care institutions in Ethiopia: a cluster randomized controlled trial
Source: Front Psychiatry. 2025 Apr 17;16:1548356. doi: 10.3389/fpsyt.2025.1548356 (PMC12043592; doi:10.3389/fpsyt.2025.1548356)
Supplement: Supplementary file 1 [file DataSheet1.zip › Data Sheet 1/Supplementary file1.pdf]

CONSORT 2010 checklist of information to include when reporting a cluster randomised trial

Section/Topic	Item No	Standard Checklist item	Extension for cluster designs	Page No *
**Title and abstract**				
	1a	Identification as a randomized trial in the title	Identification as a cluster randomised trial in the title	Title and Abstract section
	1b	Structured summary of trial design, methods, results, and conclusions (for specific guidance see CONSORT for abstracts)		Abstract section
**Introduction**				
**Background and objectives**	2a	Scientific background and explanation of rationale	Rationale for using a cluster design	Under introduction ,Study design, area, and period section
	2b	Specific objectives or hypotheses	Whether objectives pertain to the cluster level, the individual participant level or both	Under introduction ,Study design, area, and period section
**Methods**				
**Trial design**	3a	Description of trial design (such as parallel, factorial) including allocation ratio	Definition of cluster and description of how the design features apply to the clusters	Under Study design, area, and period section
	3b	Important changes to methods after trial commencement (such as eligibility criteria), with reasons		N/A
**Participants**	4a	Eligibility criteria for participants	Eligibility criteria for clusters	Under inclusion and exclusion criteria section
	4b	Settings and locations where the data were collected		Under Study design, area, and period section
**Interventions**	5	The interventions for each group with sufficient details to allow replication, including how and when they were administered	Whether interventions pertain to the cluster level, the individual participant level, or both	Under intervention description section, Supplementary file3, reference [21]
**Outcomes**	6a	Completely defined pre-specified primary and secondary outcome measures, including how and when they were assessed	Whether outcome measures pertain to the cluster level, the individual participant level or both	Under outcome measure section and Supplementary file2
	6b	Any changes to trial outcomes after the trial		N/A

		commenced, with reasons		
**Sample size**	7a	How sample size was determined	Method of calculation, number of clusters(s) (and whether equal or unequal cluster sizes are assumed), cluster size, a coefficient of Intracluster correlation (ICC or *k*), and an indication of its uncertainty	Under sample size determination and sampling procedure section
	7b	When applicable, explanation of any interim analyses and stopping guidelines		N/A
**Randomisation:**				
**Sequence generation**	8a	Method used to generate the random allocation sequence		Under randomization and blinding section
	8b	Type of randomisation; details of any restriction (such as blocking and block size)	Details of stratification or matching if used	Under randomization and blinding section
**Allocation concealment mechanism**	9	Mechanism used to implement the random allocation sequence (such as sequentially numbered containers), describing any steps taken to conceal the sequence until interventions were assigned	Specification that allocation was based on clusters rather than individuals and whether allocation concealment (if any) was at the cluster level, the individual participant level or both	Under randomization and blinding section
**Implementation**	10	Who generated the random allocation sequence, who enrolled participants, and who assigned participants to interventions	Replace by 10a, 10b and 10c	Under randomization and blinding section
	10a		Who generated the random allocation sequence, who enrolled clusters, and who assigned clusters to interventions	Under randomization and blinding section
	10b		Mechanism by which individual participants were included in clusters for the purposes of the trial (such as complete enumeration, random sampling)	Under randomization and blinding section
	10c		From whom consent was sought (representatives of the cluster, or individual cluster members, or both), and whether consent was sought before or after randomisation	Under sample size determination and sampling procedure and randomization and blinding section

**Blinding**	11a	If done, who was blinded after assignment to interventions (for example, participants, care providers, those assessing outcomes) and how		Under randomization and blinding section
	11b	If relevant, a description of the similarity of interventions		Under intervention description section,
**Statistical methods**	12a	Statistical methods used to compare groups for primary and secondary outcomes	How clustering was taken into account	Under data analysis section
	12b	Methods for additional analyses, such as subgroup analyses and adjusted analyses		Under data analysis section
**Results**				
**Participant flow (a diagram is strongly recommended)**	13a	For each group, the numbers of participants who were randomly assigned, received intended treatment, and were analysed for the primary outcome	For each group, the numbers of clusters that were randomly assigned, received intended treatment, and were analysed for the primary outcome	Under result section , and Figure 1
	13b	For each group, losses and exclusions after randomisation, together with reasons	For each group, losses and exclusions for both clusters and individual cluster members	Under result section , and Figure 1
**Recruitment**	14a	Dates defining the periods of recruitment and follow-up		Under result section, and Figure 1
	14b	Why the trial ended or was stopped		N/A
**Baseline data**	15	A table showing baseline demographic and clinical characteristics for each group	Baseline characteristics for the individual and cluster levels as applicable for each group	Under demographic variable/baseline characteristics
**Numbers analysed**	16	For each group, number of participants (denominator) included in each analysis and whether the analysis was by original assigned groups	For each group, number of clusters included in each analysis	Under data analysis, and result section , and Figure 1
**Outcomes and estimation**	17a	For each primary and secondary outcome, results for each group, and the	Results at the individual or cluster level as applicable and a coefficient of Intracluster	Under data analysis, and result

		estimated effect size and its precision (such as 95% confidence interval)	correlation (ICC or k) for each primary outcome	section
	17b	For binary outcomes, presentation of both absolute and relative effect sizes is recommended		Under result section, (PPD literacy part)
**Ancillary analyses**	18	Results of any other analyses performed, including subgroup analyses and adjusted analyses, distinguishing pre-specified from exploratory		Under result section, (PPD literacy part)
**Harms**	19	All-important harms or unintended effects in each group (for specific guidance see CONSORT for harms[1])		N/A
**Discussion**				
**Limitations**	20	Trial limitations, addressing sources of potential bias, imprecision, and, if relevant, multiplicity of analyses		Study limitation section
**Generalizability**	21	Generalizability (external validity, applicability) of the trial findings	Generalizability to clusters and/or individual participants (as relevant)	Under conclusion and recommendation section
**Interpretation**	22	Interpretation consistent with results, balancing benefits and harms, and considering other relevant evidence		Under discussion and recommendation section
**Other information**				
**Registration**	23	Registration number and name of trial registry		Under abstract section
**Protocol**	24	Where the full trial protocol can be accessed, if available		Supplementary file 1,reference (21)
**Funding**	25	Sources of funding and other support (such as supply of drugs), role of funders		Under funding section

** Note: page numbers optional depending on journal requirements*
